# Detecting anomalous experiences in the community: The Transpersonal Experiences Questionnaire (TEQ)

**DOI:** 10.1111/papt.12445

**Published:** 2023-01-09

**Authors:** Charles Heriot‐Maitland, Silia Vitoratou, Emmanuelle Peters, Karlijn Hermans, Til Wykes, Caroline Brett

**Affiliations:** ^1^ Department of Psychology, Institute of Psychiatry, Psychology & Neuroscience King's College London London UK; ^2^ Department of Biostatistics and Health Informatics, Institute of Psychiatry, Psychology & Neuroscience King's College London London UK; ^3^ South London and Maudsley NHS Foundation Trust London UK; ^4^ Department of Neurosciences, Research Group Psychiatry, Center for Contextual Psychiatry KU Leuven Leuven Belgium; ^5^ Sussex Partnership NHS Foundation Trust Worthing UK

**Keywords:** continuum, item response theory, psychosis, psychotic‐like experiences, schizotypy

## Abstract

**Objectives:**

There is growing recognition of the value of researching anomalous experiences in the general population to aid our understanding of the psychosis continuum. There are key differences in aims, foci and epistemologies of existing measures, with varying utility for specific research designs. This study addresses gaps in the literature by developing a measure of anomalous experiences with utility for longitudinal (time‐sensitive) research, and with particular reliability for people towards the upper (high scoring) end of the continuum.

**Methods:**

An online sample was recruited from the general population to provide questionnaire data for two study parts: (A) item selection and (B) psychometric evaluation. For Part A, both classical test theory and item response theory methods were used to select which items to be included from an initial pool of 57, generated from individuals with persistent anomalous experiences. For Part B, psychometric properties of the resulting measure were evaluated using exploratory and confirmatory factor analysis and tests of reliability and validity.

**Results:**

Scores were provided by 532 participants, from which a 19‐item scale, the Transpersonal Experiences Questionnaire (TEQ), was developed. The TEQ was found to be a unidimensional scale, with satisfactory internal consistency (0.85), good test–retest reliability and convergent validity.

**Conclusions:**

The TEQ can be used as a unidimensional scale to detect anomalous experiences in the general population, with particular reliability for people with higher incidence of these experiences.

**Practitioner points**

- The TEQ can be used to detect and examine anomalous experiences in the general population reliably, particularly those at the upper end of the continuum, anchored to a specific timeframe.
- The TEQ is a tool that can contribute to future research and understanding of what distinguishes clinical and non‐clinical anomalous experiences, with implications for the targeting of interventions for clinical groups.

## INTRODUCTION

Research into understanding the nature of psychotic phenomena is no longer limited to clinical populations, but is increasingly carried out in the general population, where psychotic‐like experiences exist on a continuum (van Os et al., 2009). This has led to demand for scales to detect these experiences in community samples.

In the field of psychosis research, the term *psychotic‐like experiences* (PLEs) has been widely used to differentiate these experiences in the general population from the psychotic symptoms of clinical populations. However, some researchers have preferred *anomalous experiences* (Bell et al., 2006; Brett et al., 2007) as it still captures an out‐of‐the‐ordinary quality of experience, but without pathological links and connotations. Another term, *transpersonal experience* (literally meaning experiences that go *beyond* the personal identity or self), has not been used within the psychosis research field, but is familiar terminology to those involved with the psychological study of spiritual phenomena (e.g. the field of Transpersonal Psychology; Friedman [2002]) and is consistent with the attributions of experiencers themselves, many of whom attach spiritual/mystical/psychic meaning to their experience (Heriot‐Maitland et al., 2012).

In researching these experiences, not only has the approach to terminology been varied but also has the research measurement. Some measures take clinical (psychotic) symptoms as their starting point, while others are based on schizotypal personality traits (Claridge, 1994). Symptom‐anchored measures have generally either focussed on detecting a ‘proneness’ or ‘prodrome’ to psychotic disorders in the general population or on assessing the presence of PLEs in healthy populations, therefore demonstrating the continuity—and potentially non‐pathological nature—of these experiences. Schizotypy‐anchored measures, on the other hand, are focused on assessing schizotypal personality traits normally distributed across the general population. In turn, the choice and wording of items is often dependent on what the measure is designed to achieve, with the former using similar terminology to clinical symptom scales (e.g. ‘Do you ever feel there is a conspiracy against you?’, from the Peters et al Delusions Inventory (PDI), Peters et al. (2004); ‘I have been troubled by hearing voices in my head’, from the Launay‐Slade Hallucination Scale, Launay and Slade [1981]), and the latter including milder but stable traits across a number of dimensions (e.g. ‘I tend to keep my feelings to myself’ or ‘I am an odd, unusual person’, from the Schizotypal Personality Questionnaire, Raine [1991]).

Several reviews highlight key distinctions between different approaches to schizotypy (Grant et al., 2018; Kwapil & Barrantes‐Vidal, 2015) and to the psychosis continuum more generally, positing either fully, quasi or hybrid dimensional models (Baumeister et al., 2017; Kaymaz & van Os, 2010; Toh et al., 2022; van Os & Linscott, 2012; Van Os & Reininghaus, 2016). Although a full discussion of these models is beyond the scope of this paper, a key distinction is whether (a) the abnormal state is the reference point, with the continuity being construed as varying degrees of expression in clinical signs and symptoms, or (b) the presence of anomalous experiences is disconnected from pathological framing, with illness or health outcomes representing a separate dimension, as originally proposed by Claridge (1987). The view that clinical relevance is linked to other biopsychosocial factors, rather than the presence of anomalous experiences, is supported by a body of empirical evidence (Peters et al., 2016).

Grant et al. (2018) call for researchers to clarify the framework within which their research is being conducted. The current study focuses on anomalous experiences that mimic the positive symptoms typically found in individuals with a diagnosis of psychosis, rather than multidimensional schizotypal personality traits. However, it is aligned to a theoretical stance that views such experiences as part of normal human variation, rather than being indicative of a risk factor for pathology.

Conceiving these phenomena as distinct from pathology implies that measurement should also be de‐coupled from pathology; for example, using scales where both language and item selection are based on the experience of non‐clinical populations. Our research group and others have focused on recruiting people from spiritual/mystical/psychic organisations (Brett et al., 2014; Luhrmann et al., 2019; Moseley et al., 2022; Peters et al., 2016; Powers III et al., 2016). These are individuals in the non‐clinical or non ‘need‐for‐care’ population (i.e. without psychosis‐related diagnoses or services) with persistent anomalous experiences that they describe in spiritual or transpersonal terms. In this study, the approach was to identify the experiences to be assessed, and the terminology of items, from this non‐clinical population and to use a title in keeping with the non‐pathological framing and attributions of this population: the *Transpersonal Experience Questionnaire* (TEQ).

The intention for the TEQ was to capture a broad range of experiences, across ideational and perceptual domains, and contain both positive and negative experiences (i.e. not just those linked to mental distress typically encountered in clinical services). This differentiates the TEQ from the recently published Questionnaire for Psychotic Experiences (QPE; Rossell et al., 2019), which although capturing a broad range of experience, anchors its range within clinical symptomatology. This also differentiates the TEQ from many other measures that have focused on specific domains, for example *ideational* (Peters et al Delusion Inventory, PDI [Peters et al., 1999]), *perceptual* (Cardiff Anomalous Perceptions Scale, CAPS [Bell et al., 2006]) or *sensory* phenomena (Multi‐Modality Unusual Sensory Experiences Questionnaire, MUSEQ [Mitchell et al., 2017]). With the TEQ, the aims were to de‐couple measurement from pathological framing whist still ensuring its relevance as a tool for clinical research. Also, while retaining a wide breadth of experiences was important, this study aimed to develop a measure with a limited number of items to ensure user‐friendliness.

The specific aims also differentiate the TEQ from the widely used O‐LIFE (Mason et al., 1995), which aimed to measure schizotypal traits, rather than specifically the occurrence of anomalous experiences. As a result, the wording of some items would not have been suitable, for example ‘Do you believe in telepathy’ or ‘Have you wondered whether the spirits of the dead can influence the living’. The O‐LIFE also includes items such as ‘Have you occasionally felt as though your body did not exist’ or ‘Have you felt as though your head or limbs were somehow not your own’, which tap into symptoms that are included in diagnostic scales but rarely reported by either clinical or non‐clinical groups. Therefore, the aims of the TEQ were to select items that captured a wide variety of representative anomalous experience which, although out‐of‐the‐ordinary (and still very relevant to clinical samples), are not necessarily associated with distress, pathology, or a ‘need for care’ (van Os et al., 2009).

For the current study, it was important to develop the TEQ as a scale suitable for people with high incidence of anomalous experiences, so that it could be used meaningfully in both general population and clinical samples with persistent experiences. The selection preference was towards items with good reliability among high scorers, that is those with a variety of experiences over a given period. Item Response Theory (IRT; Lord, 1980) allows the analysis of each item's reliability as an indicator of a general continuum as well as for different places along that continuum, i.e. where there are low, medium or high expressions of that continuum. Hence, for the current purposes, IRT could be used to identify items that are most useful for research into people with higher incidence of anomalous experience.

Another consideration was for the measure to capture a snapshot in time, and therefore to be anchored to a specific timeframe. Many previous measures were not designed in this way; for example, the MUSEQ (Mitchell et al., 2017) asks participants to rate whether ‘there have been times when…’ and the O‐LIFE (Mason et al., 1995) asks about the presence of experiences in general, for example ‘do you sometimes feel…’ and ‘on occasions, have you seen…’. This study aimed to develop items that could anchor experiences in time: ‘in the last 7 days’, thus making the measure useful for repeated timepoints.

In summary, the aim of this study was to establish a psychometrically robust but brief, time‐anchored measure of non‐pathologically framed anomalous experiences called the *Transpersonal Experience Questionnaire* (TEQ) that could be used by both clinical and non‐clinical populations.

## METHODS

### Sample and design

An online sample was recruited from the general population to participate in two parts of the study: Part A: item selection; and Part B: psychometric evaluation. Exclusion criteria were being under 18 years and reporting contact with mental health services for a psychosis‐related diagnosis (answering that they had a ‘diagnosis received for a psychotic disorder [e.g. schizophrenia]’).

For Part A, a sample size of 285 was estimated to be adequate for item selection purposes (i.e. minimum of five participants per item for factor analysis [Stevens, 1996]). For item selection, the psychometric properties of all items were evaluated using classical test theory methods (endorsement, test–retest reliability and internal consistency), item response theory methods (information/precision of each item), and content validity evaluations (experts' input).

Part B evaluated the factor structure and psychometric properties of the measure. To evaluate test–retest reliability, it was estimated that a sub‐sample of at least 50 would be required to repeat the measure seven days later. To evaluate convergent validity, a comparator measure was administered (O‐LIFE‐Unusual Experiences, UnEx [Mason et al., 1995]). The analysis of factor structure involved splitting participants into two sub‐samples: one for exploratory factor analysis (EFA), and one for confirmatory factor analysis (CFA). The ‘EFA sample’ was the same as the item selection sample (above) and a second, similarly sized, ‘CFA sample’ only completed the items selected for the measure.

### Measures

The *Transpersonal Experiences Questionnaire* (TEQ): As part of an unpublished PhD study (Brett, 2005), Brett and colleagues generated a pool of questionnaire items from a broad sample of individuals with anomalous experiences, comprising both those with a diagnosis of a psychotic disorder and a non‐clinical population, using the same extensive piloting, interviews, and adjusting procedures as in the Anomalous Experiences (AANEX) Interview (Brett et al., 2007). The aim was to use these questionnaire items (a total pool of 57) as the basis for the TEQ. Brett's initial pool of 57 were developed in the form of self‐rated measurements of anomalous experiences across the clinical and non‐clinical population. Item scores ranged from 1 (‘never experienced this’) to 5 (‘definitely experienced this [frequently]’). For the current study, three adaptations were made: (i) items were converted to online format; (ii) ‘In the past 7 days…’ was added to anchor to a time frame; (iii) response options were reduced to binary (Yes/No) scores to reduce respondent demand.

The *Oxford‐Liverpool Inventory of Feelings & Experiences* (Mason et al., 1995) is a 159‐item self‐rated scale measuring four dimensions of schizotypal personality in the general population. The current study used only the Unusual Experiences factor (assessing ‘positive schizotypy’), a 30‐item subscale pertaining to unusual perceptual experiences, hallucinations, and magical thinking.

### Procedure

The study was approved by the King's College London Research Ethics Subcommittee (ref: PNM/14/15‐26). Participants were recruited via adverts placed on websites and email lists, including King's College London (http://www.kcl.ac.uk) and www.experimatch.com. Adverts stated that the research was on ‘anomalous (“unusual”) experiences, such as out‐of‐the‐ordinary perceptions, feelings, or spiritual‐type experiences that are somehow different to everyday life’, however, made clear that ‘it doesn't matter whether or not you think you have these kinds of experiences’. Participation was remunerated through a prize draw (1st, 2nd and 3rd prizes), with an additional prize draw for those completing 7‐day retests. Invitations for retest were sent to the first participants entering the study until at least 50 were completed, at which point invitations ceased.

### Analysis

Multiple analysis methodologies were used. For item selection, criteria were based on content validity, classical test theory indices, item response theory (IRT) indices and exploratory factor analysis (EFA) for categorical data. The criteria are briefly described in each results section, with additional information provided in Table S1. In the refined item set, where the number of items was sufficiently reduced, the sample size was adequate (Kyriazos, 2018) to spit the data into two halves (using a random number generator) and apply exploratory factor analysis in the first half and confirmatory factor analysis (CFA) in the second. An IRT model was used in the entire sample for the final set of items.

As the data were binary, item factor analysis for categorical data (EFA and CFA) using the weighted least squares estimator (WLSMV; Muthén et al. [1997]) was applied to investigate the dimensionality of the scale. Promax rotation was used as emerging factors were assumed to be correlated (oblique). The number of factors to retain was decided based on the Guttman‐Kaiser criterion (Guttman, 1954; Kaiser, 1960), parallel analysis (Horn, 1965) for categorical data using R package ‘random.polychor.pa’ for categorical data (Presaghi & Desimoni, 2019), Cattell's (1966) scree plot, and upon investigation of the goodness of fit indices.

The item response theory, two‐parameter logistic model (2‐PL IRT) was employed to investigate the properties of each item within their dimension (factor).

For demographic analyses, the multiple indicator multiple causes model (MIMIC; Muthén, 1979) was used to assess potential measurement bias, and then non‐parametric tests (Mann–Whitney) compared TEQ scores with demographic variables.

Mplus 7 software (Muthén & Muthén, 1998–2017) was used to fit the latent trait models (IRT, EFA, and CFA) and obtain the following goodness of fit indices: the relative chi‐square (relative 𝜒^2^: values close to 2 suggest close fit; Hoelter [1983]), the Root Mean Square Error of Approximation (values below .06 indicate close fit RMSEA; Hu and Bentler [1999]), the Comparative Fit Index (CFI: values above .95 indicate a close fit; Bentler [1990]), the Taylor–Lewis Index (TLI: values above .95 are required for close fit; Hu and Bentler [1999]) and the Standardised Root Mean Residual (SRMR: values below .08 suggest a good fit; Hu and Bentler [1999]).

The remaining analyses were run in SPSS 24 (IBM, 2016). Due to the online questionnaires providing complete data sets, there was no requirement for methods of handling missing data. Items were re‐administered 7 days later to 59 individuals to investigate stability of responses (test–retest reliability; see Table S1 for details).

## RESULTS

### Characteristics of the samples

A total of 544 participants were recruited, of whom 12 were excluded for a psychosis‐related diagnosis, making a final sample of 532. Part A item selection included 283 participants. For Part B, the same 283 comprised the ‘EFA sample’ and a further 249 comprised the ‘CFA sample’. The majority were women (79%), white (white British 45% and white other 30%), in the age range 18–29 (70%). 62% per cent had never visited services for a mental health problem, 25% had received services for a mood disorder diagnosis (e.g. depression, anxiety), 4% for an ‘Other’ (non‐psychosis) diagnosis, and 9% with no diagnosis. Table 1 shows demographics for each sample.

**TABLE 1:** Participant numbers for each part of the study and demographics

	Part A item selection from initial pool of 57 items (*N* = 283)		Part B psychometric evaluation of TEQ (*N* = 532)			
			Exploratory factor analysis (*N* = 283)		Confirmatory factor analysis (*N* = 249)	
Gender						
Male	63	(22.3%)	63	(22.3%)	44	(17.7%)
Female	220	(77.7%)	220	(77.7%)	205	(82.3%)
Age group						
18–29	188	(66.5%)	188	(66.5%)	186	(74.7%)
30–39	44	(15.5%)	44	(15.5%)	30	(12.0%)
40–49	27	(9.5%)	27	(9.5%)	15	(6.0%)
50–59	14	(4.9%)	14	(4.9%)	8	(3.2%)
60+	10	(3.5%)	10	(3.5%)	10	(4.0%)
Ethnicity						
White British	130	(45.9%)	130	(45.9%)	111	(44.6%)
White other	85	(30.0%)	85	(30.0%)	74	(29.7%)
Mixed	15	(5.3%)	15	(5.3%)	18	(7.1%)
Asian	43	(15.2%)	43	(15.2%)	31	(12.4%)
Black	4	(1.4%)	4	(1.4%)	4	(1.6%)
Other	6	(2.1%)	6	(2.1%)	11	(4.4%)
Language						
English	216	(76.3%)	216	(76.3%)	193	(77.5%)
Other	67	(23.7%)	67	(23.7%)	56	(22.5%)
Education						
No degree	83	(29.3%)	83	(29.3%)	82	(32.9%)
Degree	120	(42.4%)	120	(42.4%)	83	(33.3%)
Higher degree	80	(28.3%)	80	(28.3%)	84	(33.7%)
Mental Health services						
No	176	(62.2%)	176	(62.2%)	154	(61.8%)
Yes	107	(37.8%)	107	(37.8%)	95	(38.2%)

### Part A: Item selection

Two analysis methods, (a) classical test theory and (b) item response theory, were employed in parallel with content validity checks, using independent evaluations by two authors, and further validity checks by a third author. On occasions where an item was problematic on test criteria but had particular content value (e.g. in retaining the breadth of experiences from the initial pool), the item was retained for subsequent stages of analysis. Where there was conflict between two items, content value was prioritised in the decision to retain.

#### Classical test theory

Classical test theory was used to remove items based on endorsement, stability, and internal consistency criteria. Table S1 summarises the criteria and Table S2 shows how each item fared against these. In total, 25 items were omitted at this stage. Five did not fulfil criteria but were retained due to content validity (2, 4, 13, 15, 16, marked with * in Table S2). 32 proceeded to the next stage.

#### Item response theory

In stage 2, an *item response theory* (IRT) model (2‐parameter logistic model [Lord, 1980]), was implemented to explore the precision (the *information*, in IRT terminology) of each item. As IRT models require unidimensionality, exploratory factor analysis (EFA; Wirth & Edwards, 2007) was used on the 32 items to identify the number of IRT models required (one per factor). Although a one‐factor model showed adequate fit (rel *χ* ^2^ = 1.6, RMSEA = .044, CFI = .912, TLI = .906), the 2‐factor model was better fitted (rel *χ* ^2^ = 1.3, RMSEA = .033, CFI = .955, TLI = .949).

Figures S1 and S2 show IRT results as item information curves (IFC) for the two factors. Omission decisions were made from visual analysis of these curves, ensuring no replication of items (i.e. two overlapping item curves) and ensuring that reliable (highly informative) items were present in all levels of the factor (i.e. different positions along the *x*‐axis). In line with the aims of this study, there was a particular interest in items that were reliable for high scorers (i.e. further right along the *x*‐axis). Eighteen items loaded onto Factor 1 (Figure S1), of which seven were omitted (2, 13, 19, 29, 46, 49, 57). Fourteen loaded onto Factor 2 (Figure S2), of which six were omitted (3, 5, 9, 11, 17, 18). The remaining 19 items comprised what will hereafter be referred to as the TEQ (Appendix A).

### Part B: Psychometric evaluation of TEQ

Participants were split into two samples to examine the factor structure: (i) ‘EFA sample’ (*n* = 283) and (ii) ‘CFA sample’ (*n* = 249). Samples were then combined (*n* = 532) to examine (iii) reliability and validity of TEQ and (iv) difficulty and discrimination of individual items. Finally, (v) demographic characteristics of the combined sample in relation to TEQ scores were investigated.

#### Exploratory factor analysis of TEQ

In the sample correlation matrix, there were four eigenvalues above 1 (8.545, 1.966, 1.263, 1.019). Both a 1‐factor (rel *χ* ^2^ = 1.4, RMSEA = .040, CFI = .935, TLI = .927) and a 2‐factor (rel *χ* ^2^ = 1.2, RMSEA = .027, CFI = .0973, TLI = .966) model provided close fit to the data, indicating that no further factors should be extracted (overfitting). Parallel analysis indicated that one factor should be retained. The scree plot (Figure S3) also suggested the extraction of only one factor.

#### Confirmatory factor analysis of TEQ

Confirmatory factor analysis suggested a close fit for both the 1‐factor (rel *χ* ^2^ = 1.41, RMSEA = .041, CFI = .955, TLI = .950) and 2‐factor (rel *χ* ^2^ = 1.27, RMSEA = .033, CFI = .972, TLI = .968) models. Therefore, for additional information on the scale's dimensionality, the bifactor model approach was used, as recommended by Reise et al. (2007). The Reise et al. (2007) method compares the loadings of three models: (a) the unidimensional model (containing only a general factor, onto which all items load), (b) the multidimensional model of specific factors (here, two factors) and (c) the combination of (a) and (b), the bifactor model. If the general factor presence causes the loadings on specific factors to become non‐salient, evidence of unidimensionality is provided. If loadings on the general factor become negligible in the presence of specific factors, multidimensionality is supported. In this case, the fit of the bifactor model was close (rel *χ* ^2^ = 1.1, RMSEA = .021, CFI = .990, TLI = .987) and in the presence of a general factor, loadings on the two factors were substantially reduced, becoming non‐significant in most cases (Table S3).

Combining evidence from EFA, parallel analysis, and CFA (model fit indices and bifactor loadings), we conclude that TEQ is a unidimensional scale, which is in line with greater content coherence for one‐ compared with two‐factor structures. Therefore, the TEQ was considered unidimensional for the remaining analyses.

#### Psychometric properties (reliability and validity) of TEQ

Item endorsement varied from 4% (item 15) to 29% (item 5; see Table 2). Cronbach's alpha was satisfactory (.85), and no item was found to reduce the internal consistency of the scale. The item‐total correlations ranged from .35 to .52, further confirming internal consistency. The items retained in the final scale had at least 83% agreement between the two time points (specifically 83%–97%), showing good test–retest reliability. The total TEQ score was highly correlated with O‐LIFE‐UnEx scores (Spearman's rho = .74, *p* < .001), indicating convergent validity.

**TABLE 2:** EFA/CFA loadings, IRT parameters and reliability indices for TEQ items

Item	Short description	Endorsement	Loadings^a^		IRT parameters^a^		Reliability			
							Internal consistency		Stability (test–retest)	
		*N* (%)	EFA	CFA	a	b	AID	ITC	*k*	%
TEQ01	In contact	75 (14%)	0.65	1.11	2.14	^−^0.59	0.84	0.43	0.52	89.8
TEQ02	Seeing	30 (6%)	0.59	1.02	^+^2.32	0.99	0.84	0.35	0.13	86.4
TEQ03	Others read thoughts	73 (14%)	0.71	0.93	1.64	0.93	0.84	0.44	0.54	94.9
TEQ04	Smelling	99 (19%)	0.54	0.66	1.56	0.71	0.84	0.36	0.41	81.4
TEQ05	Thoughts whirl	155 (29%)	0.52	0.88	^−^0.93	0.74	0.84	0.40	0.10	96.6
TEQ06	‘Mission’ revealed	54 (10%)	0.68	0.94	1.85	1.00	0.84	0.42	0.48	86.4
TEQ07	Body sensations	84 (16%)	0.61	1.26	1.44	1.01	0.83	0.48	0.48	96.6
TEQ08	Messages or hints	118 (22%)	0.76	1.24	1.05	1.14	0.83	0.52	0.35	89.8
TEQ09	Picking up thoughts	87 (16%)	0.70	1.23	1.44	0.97	0.83	0.46	0.66	91.5
TEQ10	Monitored	73 (14%)	0.67	1.51	1.45	1.22	0.83	0.51	0.40	91.5
TEQ11	Others' emotions	55 (10%)	0.72	1.01	1.81	1.02	0.84	0.43	0.62	91.5
TEQ12	Isolated	104 (20%)	0.71	0.91	1.27	0.95	0.83	0.47	0.37	94.9
TEQ13	Caused event	37 (7%)	0.67	1.47	1.89	1.35	0.84	0.46	0.78	96.6
TEQ14	Time disorientation	103 (19%)	0.65	1.09	1.23	1.03	0.83	0.48	0.64	89.8
TEQ15	Bodily movements	19 (4%)	0.84	1.18	2.29	^+^1.37	0.84	0.40	0.55	84.7
TEQ16	Influenced by others	41 (8%)	0.70	0.77	2.11	0.96	0.84	0.38	0.35	86.4
TEQ17	Loss of identity	77 (14%)	0.78	1.05	1.45	1.13	0.83	0.50	0.47	88.1
TEQ18	Hearing	47 (9%)	0.65	0.85	2.05	0.92	0.84	0.39	0.40	83.1
TEQ19	Events in reference	79 (15%)	0.56	0.81	1.70	0.80	0.84	0.39	0.57	89.8

*Note*: +, − in column a denote the most and least difficult (to endorse) item of the factor, and in column b denote the most and least discriminative item.

Abbreviations: a, difficulty parameter; AID, alpha if item deleted (complete sample); b, discrimination parameter; CFA, Confirmatory factor analysis (sample 2); EFA, exploratory factor analysis (sample 1); IRT, Item response theory model (complete sample); ITC, item total correlation (complete sample); k, Cohen's kappa % agreement (test–retest sample); *N*, number of positive responses.

^a^ All loadings were significant (*p* < .05).

#### Psychometric properties (difficulty, discrimination, and precision) of individual TEQ items

Table 2 presents the difficulty and discrimination parameters, with corresponding Item Characteristic Curves (ICCs) and Item Information Curves (IFCs) shown in Figure S4. The most difficult (to endorse) question was item 2, the least difficult was item 5, and the most discriminative across different levels of the continuum was item 15. The 19 items of the TEQ have increased precision when it comes to individuals with total scores at 1 to 3 standard deviations above the mean (shown visually in Figure S4), meaning that the TEQ, as a whole, is able to reliably identify people at the higher end of the continuum.

#### Demographic characteristics

The MIMIC model was used to investigate potential measurement bias with respect to age, gender, education (up to secondary school vs. higher education), first language (English vs. other), ethnicity (white vs. other). All covariates were introduced in the model simultaneously, so results reflect the effect of each covariate adjusted for all others. Gender, language and ethnicity did not have significant effects on item endorsement, demonstrating measurement invariance (Table 3). Age significantly affected the probability of endorsing 7 items (1, 5, 6, 7, 8, 11, 17), with strong positive effect sizes in all cases apart from one (5) with a negative effect. Education also significantly affected the probability of endorsing 7 items (4, 7, 10, 12, 13, 14, 18), with strong positive effect sizes in all cases. As strong effects in the measurement (bias) of 35% of items existed in each case, it is advisable to not compare TEQ scores within age and education groups.

**TABLE 3:** Measurement invariance assessment: Significant direct effects for TEQ items (MIMIC model adjusted for age, gender, ethnicity, language, education)

Item	Direct effect	*p* Value	Effect size^a^
Age			
TEQ01	0.41	<.001	3.14
TEQ05	−0.18	.024	1.80
TEQ06	0.19	.041	1.60
TEQ07	0.21	.014	1.67
TEQ08	0.30	.001	2.20
TEQ11	0.26	.009	1.80
TEQ17	0.42	<.001	2.57
Education			
TEQ04	0.58	<.001	6.42
TEQ07	0.64	.002	5.07
TEQ10	0.96	<.001	6.31
TEQ12	0.48	.011	4.17
TEQ13	0.88	.006	4.52
TEQ14	0.63	.001	5.35
TEQ18	0.62	.005	4.57

^a^ Effect size computed as the direct effect divided by the standard deviation of the factor indicator.

The remaining demographic variables (gender, ethnicity, language and mental health services) were analysed using non‐parametric tests due to skewed TEQ data. There were no significant differences between gender (Mann–Whitney *U* = 23,260, *p* = .706), ethnicity (Mann–Whitney *U* = 25,971.5, *p* = .774) or language (Mann–Whitney *U* = 23,924, *p* = .399) groups. Those who had visited mental health services had higher scores than those who had not (Mann–Whitney *U* = 26,094.5, *p* < .001).

## DISCUSSION

### Summary of results

The final 19‐item TEQ incorporated a broad range of experiences, from ‘seeing things’, through ‘messages or hints’, to ‘time disorientation’. The final items showed good psychometric properties, good internal consistency and factor analyses confirmed it is a unidimensional scale. TEQ also has good reliability and validity as a measure of anomalous experiences in the general population. The IRT analysis demonstrated that the 19 items have particularly good precision for individuals scoring highly on the TEQ, making it suitable for detecting and researching anomalous experiences in people at the higher end of the continuum.

### Psychometric profile and potential uses

The unidimensionality of the scale is in contrast to the AANEX‐Inventory Brett et al. (2015), which showed a five‐component structure of anomalous experiences (meaning/reference; paranormal/hallucinatory; cognitive/attention; dissociative/perceptual; first‐rank symptoms). This difference may be partly due to different aims and analysis methods (Brett et al. used Principal Components Analysis) but may also have been influenced by different samples recruited. In Brett et al.'s study, almost two thirds were recruited from clinical services, which might explain the emergence of components that are more uniquely clinical, particularly in the cognitive/attention domain. Closer examination of how the TEQ items mapped onto Brett et al.'s analysis showed that the TEQ included items that represent components more prevalent in their non‐clinical group and excluded those more prevalent in their clinical groups. For example, there were no TEQ items relating to the cognitive/attention domain, which was found to be higher in the clinical groups using AANEX‐Inventory Brett et al. (2015). This is understandable given the non‐pathological focused aims and non‐clinical sampling in this study.

As a scale with measurement precision at the upper end of the continuum, the TEQ potentially has research utility for studies comparing clinical versus non‐clinical groups. For example, it could be used as for screening clinical and non‐clinical groups with similar anomalous experiences. Such comparison studies are useful in examining associated psychological, social and emotional factors (Peters et al., 2016). Since items are anchored to a timeframe, the TEQ can be used at repeated timepoints, with the potential to be used in longitudinal research designs investigating causal relationships. However, as this is a cross‐sectional study, further research is required to assess the TEQ's sensitivity to change and suitability for longitudinal research.

### Individual item profiles

There were interesting findings in terms of endorsement rates for TEQ items that mapped most closely onto the common ‘positive symptoms’, that is hallucinations (visual‐ item 2; auditory‐ item 18; olfactory‐ item 4) and delusions (paranoid‐ item 10; personal reference‐ item 19). Of the hallucination‐type items, the highest endorsement was olfactory (15.5%), then auditory (8.1%) and visual (5.3%). With the delusion‐type experiences, 12.7% had experienced ‘feeling watched or monitored’ in the past 7 days, and 13.4% had experienced ‘personal reference’. Considering this was a non‐psychosis population, these figures are relatively high. Studies of paranoid thoughts in the general population have reported similarly high prevalence, with 19% having the thought ‘I might be being observed or followed’ at least weekly (Freeman et al., 2005). However, these reports were not anchored to a specific 1‐week period, unlike the current study. Epidemiological research has reported considerably lower prevalence, with only 5.2% life‐time prevalence of hallucinatory experiences and 1.3% for delusional experiences (McGrath et al., 2015). The higher prevalence of both types of experience found in this study, in a shorter timeframe (7 days), may reflect the sensitivity of the TEQ to detecting anomalous experiences. However, an alternative explanation may be that these results were due to the self‐selecting nature of the online cohort, which may have attracted respondents with high rates of anomalous experiences.

Interestingly, the above symptom‐related items are by no means the most discriminatory (i.e. able to discriminate between people with different levels of anomalous experience). Item 10 (‘feeling watched and monitored’) is one of the more discriminatory items, although still not as discriminatory as items 15 (‘bodily movements being controlled’) and 13 (‘events caused with your mind’). Item 18 (‘hearing voices’) is one of the least discriminatory items (15th of 19), which is noteworthy since hearing voices is found to be the most prevalent symptom among schizophrenia patients (WHO, 1973), occurring in around 59% with this diagnosis (Waters et al., 2014). This reinforces the theoretical stance (Claridge, 1987) that the construct being detected is not necessarily linked to pathology. If it were, one might expect that the major identifiers of this pathology would also emerge as important identifiers and discriminators of its ‘severity’ in the population.

### Demographic profiles

The demographic results showed no significant relationships between TEQ scores and gender, ethnicity or first language. Compared with a meta‐analysis of likelihood of psychotic experience for different demographics (Linscott & van Os, 2013), these results are at odds regarding gender (the meta‐analysis found greater likelihood among men), but consistent regarding ethnicity. The discrepancy with gender may be related to the sample recruited; the majority of whom were women (79%).

Age and education were excluded from TEQ comparisons due to variability between items. Older age increased the chance of endorsing six items (1, 6, 7, 8, 11, 17), and younger age, the chance of endorsing one (5). Higher education increased the chance of endorsing seven items (4, 7, 10, 12, 13, 14, 18). Among items more likely endorsed by older ages are those with spiritual/psychic themes, which may reflect an effect of recruitment sources, in that older participants were potentially more likely recruited from non‐student communities (e.g. spiritual communities). This could not be tested as recruitment source was not collected but would be interesting to study in future. The more highly educated participants were perhaps more likely to have come from student communities (already in higher education), and the items more likely to be endorsed by them include symptom‐related items: ‘smelling’ and ‘hearing voices’ (mapping on to hallucinations), and ‘feeling watched and monitored’. Again, recruitment sources may play a role, with non‐student participants potentially coming from communities with established spiritual framings of anomalous experiences, possibly with existing practices that intentionally hone certain aspects of experience.

The other significant demographic difference was contacting mental health services for non‐psychosis‐related difficulties. This is consistent with literature linking PLEs with distress more broadly (Wusten et al., 2018). Also, in this sample, the rate of diagnosis for mood‐related conditions is above average (25% with a mood disorder vs. 17% in the general population [McManus et al., 2016]). This could be due to the age and gender bias in this sample, as prevalence of mental health problems is elevated among women (21%), particularly young women (26% for 16–24 year olds; McManus et al., 2016). The over‐representation of people with anxiety or depression is likely to influence the prevalence of anomalous experiences given these are more common among people with mood disorders (Varghese et al., 2011).

### Limitations

One limitation is that the TEQ was developed with binary (yes/no) scoring, which provides no information on other dimensions, such as the quality or severity of this experience. A high TEQ score therefore indicates a larger number of items endorsed; so, in this case, the ‘upper end’ of the continuum refers to a greater *range* of experiences, as opposed to **a greater** *severity* of experience (e.g. intensity, frequency, duration). Hence, someone with a range of ‘low level’ or mild anomalous experiences would score higher than someone with a fewer number of ‘high level’ anomalous experiences. If future studies were interested in severity of experiences, which is often used to signal clinical relevance, this would require additional scales or dimensions. Other binary measures (Peters et al., 1999) have included secondary dimensions (e.g. distress level 1–5), so this may be a consideration for future TEQ developments.

Another limitation is online recruitment, which can make it harder to ensure data quality. This study attempted to control quality by keeping the questionnaires short and giving participants expected completion times; however, there was no system of monitoring the time spent responding. Furthermore, questionnaires in general, but particularly those administered online, do not afford the opportunity to check the understanding and interpretation of items by respondents. A possible confounder not tested in this study was use of substances, which could have influenced some responses, and future studies may consider that as an exclusion criterion.

Another limitation of online questionnaires is potential for sampling selection bias, in that respondents are only those who (i) have internet access, (ii) visit websites where research studies are advertised and (iii) decide to participate. Furthermore, the test–retest reliability sub‐sample was limited to respondents who engaged with the study more than once. Future research should investigate reliability with more representative (ideally, randomised) samples and further assess validity with indices not included here, for example discriminant and incremental validity. Finally, the narrow demographic range (young, female) limits the generalisability of the results and combined with broader limitations to the external validity of online methods, these results are only applicable to the current sample.

## CONCLUSIONS

This study has developed and evaluated a reliable and valid tool for measuring non‐pathologically framed anomalous experiences in the general population, anchored to a specific timeframe. The TEQ is a unidimensional scale and has particular utility among those who have a high prevalence of experiences, which is its most unique and useful contribution to the literature. Limitations are outlined, and future research should determine whether the TEQ is sensitive to change and shows similar promising psychometric properties in populations across the psychosis continuum.

## AUTHOR CONTRIBUTIONS

**Charles Heriot‐Maitland:** Conceptualization; data curation; formal analysis; funding acquisition; investigation; methodology; project administration; writing – original draft. **Silia Vitoratou:** Formal analysis; methodology. **Emmanuelle Peters:** Conceptualization; funding acquisition; investigation; methodology; supervision; writing – review and editing. **Karlijn Hermans:** Formal analysis; methodology. **Til Wykes:** Conceptualization; funding acquisition; investigation; methodology; supervision; writing – review and editing. **Caroline Brett:** Conceptualization; methodology; writing – review and editing.

## CONFLICT OF INTEREST

None.

## Supporting information


Appendix S1.

## Data Availability

The data that support the findings of this study are available from the corresponding author upon reasonable request.

## APPENDIX A

### Transpersonal Experiences Questionnaire (TEQ)

Please read the following questions and select the response which you feel is the most accurate one for you in the past 7 days.

For each question please select either Yes or No.

### IN THE PAST 7 DAYS…

**Table:**

1. Have you had the experience of suddenly feeling as if you were in contact with someone who is not physically present, or knowing what they were thinking or feeling?	Yes	No
2. Have you had the experience of seeing something that other people could not see, or that you later found out was not there?	Yes	No
3. Have you had the experience of your thoughts being read or picked up by other people?	Yes	No
4. Have you had the experience of smelling something that other people are not aware of, or that is only perceptible to you?	Yes	No
5. Have you had the experience of thoughts rushing very rapidly through your mind, so that one idea after another comes into your head and the thoughts seem to whirl around beyond your control?	Yes	No
6. Have you had the experience of some kind of ‘mission’ or duty being revealed to you, and knowing that you have to fulfil this mission, or feeling compelled to do so?	Yes	No
7. Have you had experiences of unusual sensations in your body, not created by any obvious physical cause, for example of heat or cold, energy moving, or something entering or passing through your body?	Yes	No
8. Have you had experiences in which things in the world around you seemed to contain messages or hints, perhaps in a metaphorical or symbolic way?	Yes	No
9. Have you had the experience of picking up on other people's thoughts?	Yes	No
10. Have you had the experience of feeling monitored or watched, or otherwise the subject of external attention, when there is no obvious cause for this?	Yes	No
11. Have you had the experience of feeling emotions or thinking thoughts that were actually those of other people?	Yes	No
12. Have you experienced being in a state in which you felt cut off or isolated from things and people around you, perhaps as if there were some invisible barrier around you that prevented a normal connection?	Yes	No
13. Have you had the experience of observing an event happen and feeling as though you had caused it with your mind?	Yes	No
14. Have you had the experience of disorientation in time, so that for example, the past and the future seem distant or unavailable, and the present moment dominates, or time seems to lose its meaning?	Yes	No
15. Have you experienced your bodily movements being controlled by someone or something outside of you?	Yes	No
		
16. Have you had an experience of having your thoughts, feelings or movements influenced by other people's thoughts or gestures?	Yes	No
17. Have you had an experience of a loss of your individual identity and a sense of being part of some greater whole that extends far beyond you?	Yes	No
18. Have you had the experience of hearing things, like voices talking, when there has not been anyone around?	Yes	No
19. Have you had the experience of feeling as though events in your environment, such as the actions or comments of other people, are in reference to you, or are directed at you, even though you know that this is unlikely?	Yes	No
