# Supplementary material for: Detecting anomalous experiences in the community: The Transpersonal Experiences Questionnaire (TEQ)
Source: Psychol Psychother. 2023 Jan 9;96(2):383–98. doi: 10.1111/papt.12445 (PMC10952405; doi:10.1111/papt.12445)
Supplement: Supplementary file 1 — Appendix S1. [file PAPT-96-383-s001.docx]

**Supporting Information – Transpersonal Experiences Questionnaire**

**Table S1.** Additional information about analysis methodologies

| Part A: item selection |
| --- |
| 1. *Classical test theory*   ‘Endorsement’ criteria used:  Items with less than 10% frequency of endorsement were considered potentially problematic.  ‘Stability’ criteria used:  Since our data were skewed, and the Landis and Koch (1977) interpretation of Kappa’s values could be misleading (Viera & Garrett, 2005), we relied on the percentage agreement between test-retest time points as the best indicator of stability. Therefore, Items with less than 80% of agreement were considered potentially problematic.  ‘Internal consistency’ criteria used:  The contribution of each item to the consistency of the scale was evaluated using the item-total correlations, with values less than 0.35 been considered problematic. |
| 1. *Item response theory*   An *item response theory* (IRT) model (2-parameter logistic model, e.g. Lord (1980), was used. Unlike the classical test theory approach, the IRT model allows evaluations of the reliability of each item in relation to the construct/continuum being measured. So in this case, we are able to identify not only if an item is a reliable indicator of the ‘anomalous experience’ continuum, but at which level of the continuum the item is more reliable. For instance, if item is reliable for high scorers, but not for low scorers, and vice versa. |
| Part B: Psychometric evaluation of TEQ |
| Exploratory factor analysis of TEQ Exploratory item factor analysis for categorical data (EFA) (Wirth & Edwards, 2007) was used to identify the factor structure of the TEQ using the responses of the ‘exploratory sample’ (n=283 with complete item set). EFA is conducted using the sample correlation matrix, that is, the bivariate correlations, across all items. EFA is a method of reproducing the sample correlation matrix, allowing for stochastic errors. Kaiser’s criterion refers to the eigenvalues of the sample correlation matrix, and it is shown that the number of factors is close to the number of the eigenvalues. |
| Confirmatory factor analysis of TEQ Confirmatory factor analysis for categorical data (CFA) (Muthén, 1984) was used. Model fit was evaluated using the following indices: the relative chi-square (rel χ^2^: with preferred values close to 2; (Hoelter, 1983)), the Root Mean Square Error of Approximation (RMSEA, with preferred values less than 0.8; (Browne & Cudeck, 1993)), the Taylor-Lewis Index (TLI, with preferred values higher than 0.9; (Bentler & Bonett, 1980)), and the Comparative Fit Index (CFI, with preferred values higher than 0.9; (Bentler, 1990)). |
| 1. *Psychometric properties (reliability and validity) of TEQ*   The test-retest reliability indices of TEQ were available from Part 1 (‘stability’ analysis), because all 59 participants who did seven-day retests were among the first to enter the study.  The TEQ’s internal consistency was evaluated using Cronbach’s (1951) alpha coefficient for the entire scale, and item-total correlations for each item separately.  To evaluate convergent validity, we used a second comparator measure that has been widely used in the literature to measure anomalous experiences (O-LIFE-UnEx, Mason et al (1995)). |
| 1. *Demographic characteristics*   Evaluations of the relationships with demographic characteristics (e.g. age, gender, education) were tested by nonparametric methods due to the TEQ total scores being skewed (namely, Mann–Whitney independent samples’ test, Kruskal-Wallis ANOVA, and Spearman’s rho correlation coefficient). |
| 1. *Psychometric properties (difficulty and discrimination) of individual TEQ items*   A final IRT analysis of the 19 items was performed with the two samples combined (N=532). In addition to estimating the information (precision, reliability) of each item, the IRT model also estimates the two parameters of a) difficulty, and b) discrimination for each item. The difficulty parameter corresponds to the level (i.e. total score) required for 50% endorsement of an item. The higher the difficulty parameter, higher levels of anomalous experience are required to endorse the item. The discrimination parameter corresponds to the change in the probability of endorsement, as the level of anomalous experience increases. The higher the discrimination parameter, the more capable an item to discriminate between individuals with different levels of anomalous experience. The two IRT parameters were used to gain insight into the specificity of each item. |

**Table S2.** Reliability indices for the initial pool of 57 items (N=283)

| **item** | **Endorsement^1^** | **Stability^1^ (test-retest)** | | **Internal**  **consistency^1^** | **Item Omitted via:** | | **TEQ item** | |
| --- | --- | --- | --- | --- | --- | --- | --- | --- |
|  | **%**  **frequencies** | **Kappa** | **%**  **agreement** | **Item-Total**  **Correlation** | **CTT** | **IRT** | **Number** | **Abbreviation** |
| 1 | 15.9 | 0.52 | 89.8 | 0.47 |  |  | TEQ1 | in contact |
| 2 | 27.9 | 0.58 | 84.7 | 0.56 |  | yes |  |  |
| 3 | 22.6 | 0.57 | 89.8 | 0.43 |  | yes |  |  |
| 4 | 33.9 | 0.38 | 72.9 | 0.31 | yes |  |  |  |
| 5 | 15.9 | 0.4 | 88.1 | 0.47 |  | yes |  |  |
| 6* | 5.3 | 0.37 | 94.9 | 0.34 |  |  | TEQ2 | seeing |
| 7 | 32.2 | 0.23 | 69.5 | 0.27 | yes |  |  |  |
| 8 | 14.8 | 0.78 | 96.6 | 0.45 |  |  | TEQ3 | others read thoughts |
| 9 | 11 | 0.35 | 86.4 | 0.50 |  | yes |  |  |
| 10* | 15.5 | 0.64 | 89.8 | 0.34 |  |  | TEQ4 | smelling |
| 11 | 13.8 | 0.47 | 88.1 | 0.49 |  | yes |  |  |
| 12 | 49.5 | 0.5 | 74.6 | 0.51 | yes |  |  |  |
| 13 | 10.2 | 0.78 | 96.6 | 0.46 |  | yes |  |  |
| 14 | 4.6 | 0.85 | 98.3 | 0.44 | yes |  |  |  |
| 15 | 34.3 | 0.29 | 74.6 | 0.47 | yes |  |  |  |
| 16 | 8.5 | 0.74 | 94.9 | 0.42 | yes |  |  |  |
| 17 | 15.9 | 0.42 | 86.4 | 0.41 |  | yes |  |  |
| 18 | 33.2 | 0.54 | 81.4 | 0.42 |  | yes |  |  |
| 19 | 25.1 | 0.52 | 84.7 | 0.58 |  | yes |  |  |
| 20 | 31.8 | 0.42 | 72.9 | 0.38 | yes |  |  |  |
| 21 | 24.7 | 0.55 | 84.7 | 0.45 |  |  | TEQ5 | thoughts whirl |
| 22 | 5.7 | 0.37 | 94.9 | 0.31 | yes |  |  |  |
| 23 | 9.2 | 0.4 | 91.5 | 0.40 | yes |  |  |  |
| 24 | 10.2 | 0.35 | 86.4 | 0.44 |  |  | TEQ6 | 'mission' revealed |
| 25 | 8.1 | -0.07 | 86.4 | 0.39 | yes |  |  |  |
| 26 | 13.4 | 0.47 | 88.1 | 0.45 |  |  | TEQ7 | body sensations |
| 27 | 7.4 | 0.81 | 96.6 | 0.45 | yes |  |  |  |
| 28 | 21.9 | 0.4 | 83.1 | 0.56 |  |  | TEQ8 | messages or hints |
| 29 | 25.4 | 0.44 | 83.1 | 0.50 |  | yes |  |  |
| 30 | 16.3 | 0.57 | 89.8 | 0.48 |  |  | TEQ9 | picking up thoughts |
| 31 | 12.7 | 0.13 | 86.4 | 0.43 |  |  | TEQ10 | monitored |
| 32 | 5.7 | 0.24 | 91.5 | 0.29 | yes |  |  |  |
| 33 | 8.5 | -0.02 | 94.9 | 0.46 | yes |  |  |  |
| 34 | 11.7 | 0.54 | 94.9 | 0.45 |  |  | TEQ11 | others’ emotions |
| 35 | 20.1 | 0.41 | 81.4 | 0.53 |  |  | TEQ12 | isolated |
| 36 | 30.4 | 0.38 | 78 | 0.40 | yes |  |  |  |
| 37* | 6 | 0.10 | 96.6 | 0.41 |  |  | TEQ13 | caused event |
| 38 | 16.3 | 0.48 | 86.4 | 0.50 |  |  | TEQ14 | time disorientation |
| 39 | 8.5 | -0.05 | 89.8 | 0.36 | yes |  |  |  |
| 40* | 3.5 | 0.48 | 96.6 | 0.39 |  |  | TEQ15 | bodily movements |
| 41 | 16.6 | 0.35 | 86.4 | 0.30 | yes |  |  |  |
| 42 | 9.5 | 0.16 | 88.1 | 0.40 | yes |  |  |  |
| 43* | 6 | 0.35 | 89.8 | 0.36 |  |  | TEQ16 | influenced by others |
| 44 | 2.1 | 0.05 | 94.9 | 0.34 | yes |  |  |  |
| 45 | 8.8 | 0.25 | 91.5 | 0.46 | yes |  |  |  |
| 46 | 15.5 | 0.51 | 89.8 | 0.45 |  | yes |  |  |
| 47 | 15.9 | 0.66 | 91.5 | 0.54 |  |  | TEQ17 | loss of identity |
| 48 | 6.7 | -0.04 | 93.2 | 0.39 | yes |  |  |  |
| 49 | 24.7 | 0.34 | 81.4 | 0.47 |  | yes |  |  |
| 50 | 8.1 | 0.4 | 91.5 | 0.37 |  |  | TEQ18 | hearing |
| 51 | 13.4 | 0.62 | 91.5 | 0.36 |  |  | TEQ19 | events in reference |
| 52 | 24 | 0.19 | 71.2 | 0.41 | yes |  |  |  |
| 53 | 8.8 | 0.24 | 91.5 | 0.38 | yes |  |  |  |
| 54 | 6.7 | 0.24 | 91.5 | 0.40 | yes |  |  |  |
| 55 | 3.2 | 0.66 | 98.3 | 0.24 | yes |  |  |  |
| 56 | 8.8 | 0.88 | 98.3 | 0.46 | yes |  |  |  |
| 57 | 14.1 | 0.26 | 86.4 | 0.40 |  | yes |  |  |
| ^1^Criteria used for endorsement, stability, internal consistency are given in Table S1 section on classical test theory  *item did not fulfil criteria but was retained due to content validity | | | | | | | | |

**Figure S1.** IRT item information curves for items loading on Factor 1

**
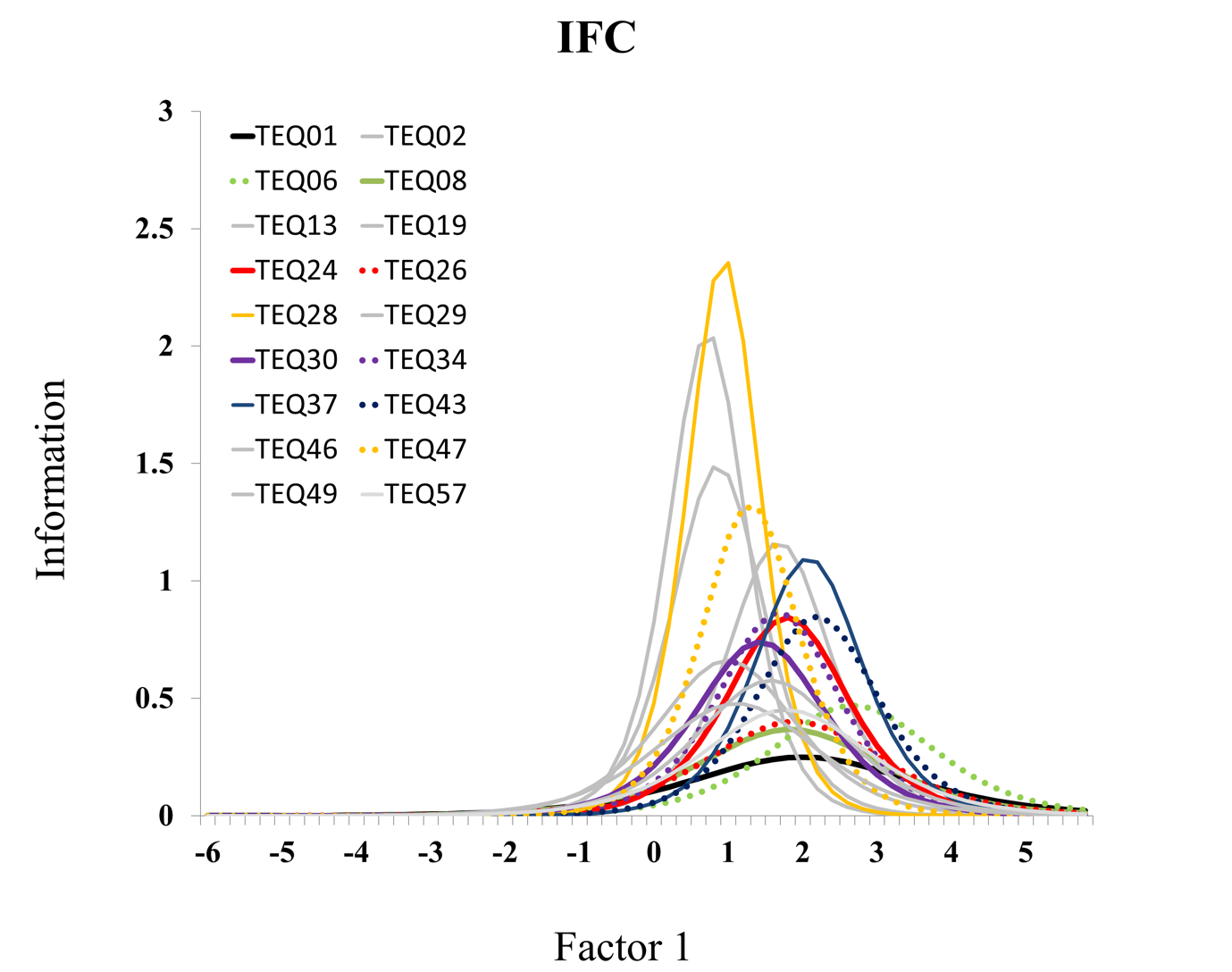
**

**Figure S2.** IRT item information curves for items loading on Factor 2

**
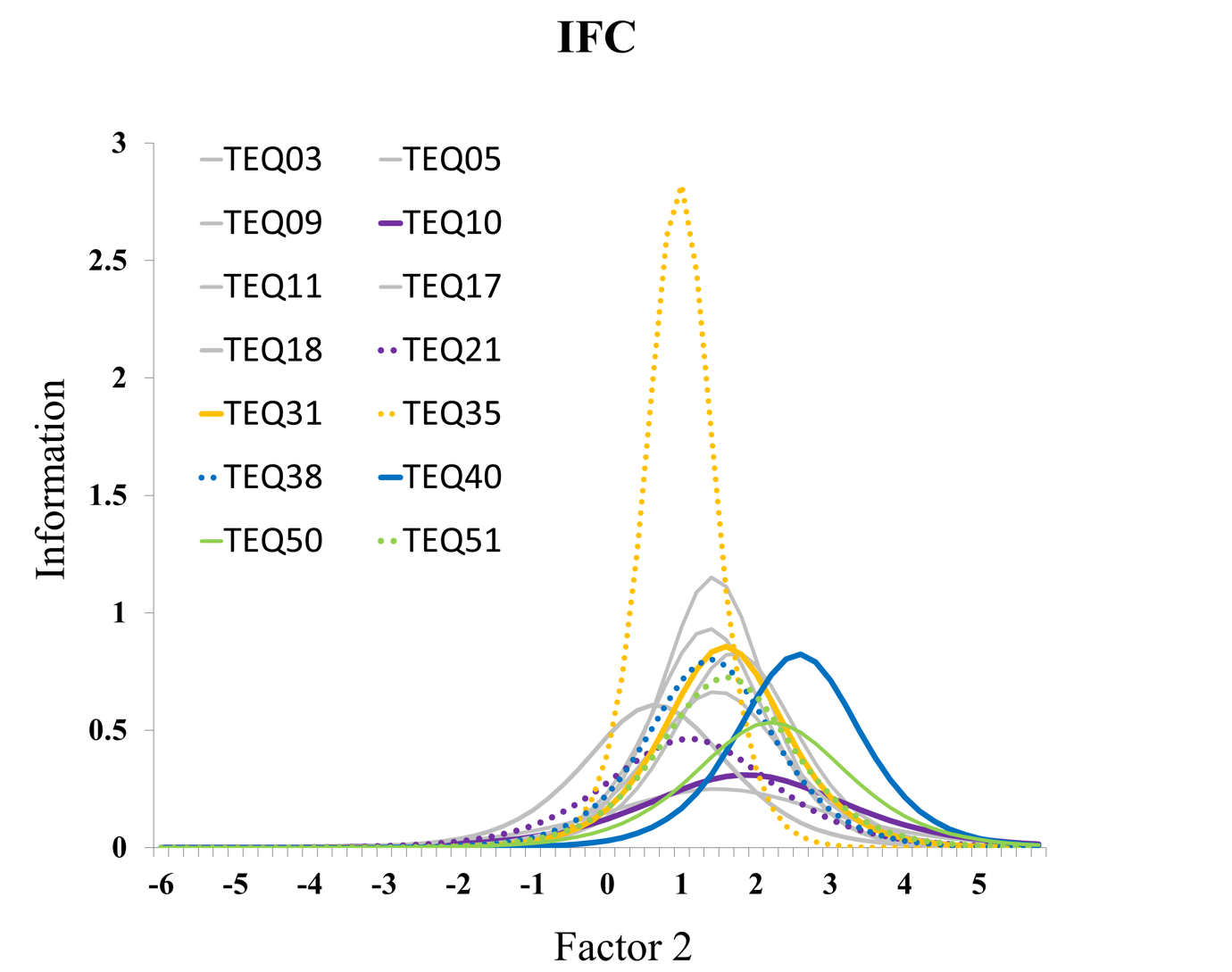
**

**Figure S3.** Scree plot for exploratory factor analysis


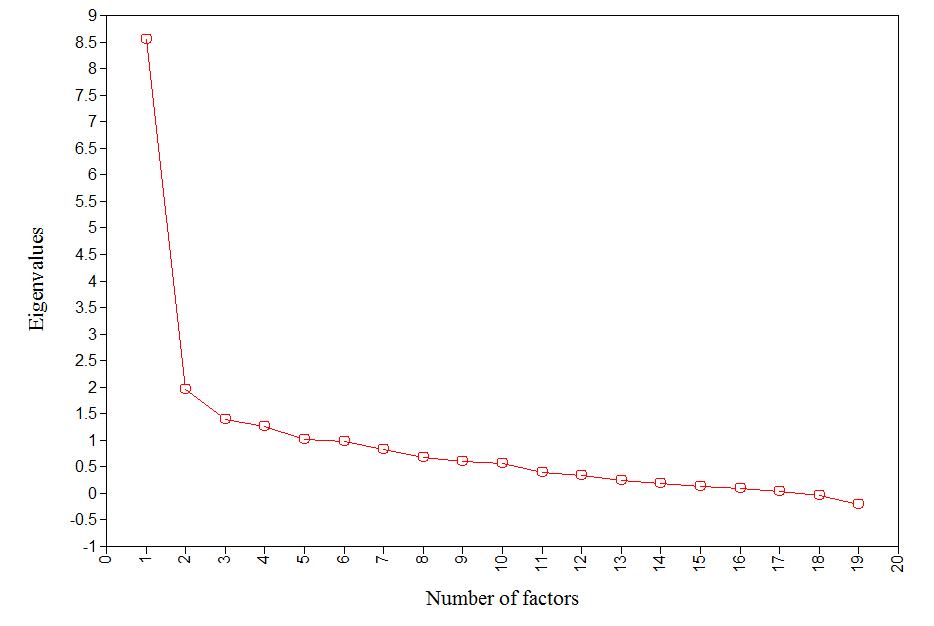


**Table S3.** Loadings for the one, two, and bi-factor two factor models

| **Item** |  | **1-factor** | |  | **2-factor** | | | |  | **Bi-factor 2-factor** | | | | | |
| --- | --- | --- | --- | --- | --- | --- | --- | --- | --- | --- | --- | --- | --- | --- | --- |
|  |  | **(Μ_1_)** | |  | **(Μ_2_)** | | | |  | **(Μ_3_)** | | | | | |
|  |  | **F1** | |  | **F1** | | **F2** | |  | **General** | | **F1** | | **F2** | |
|  |  | **λ** | **p** |  | **λ** | **p** | **λ** | **p** |  | **λ** | **P** | **λ** | **p** | **λ** | **P** |
| TEQ01 |  | 1.11 | <.01 |  | 1.22 | <.01 |  |  |  | 1.06 | <.01 | 1.26 | <.01 |  |  |
| TEQ02 |  | 1.02 | <.01 |  |  |  | 1.1 | <.01 |  | 1.32 | .01 |  |  | -.45 | .32 |
| TEQ03 |  | .93 | <.01 |  | 1.01 | <.01 |  |  |  | .86 | <.01 | .51 | .01 |  |  |
| TEQ04 |  | .66 | <.01 |  |  |  | .70 | <.01 |  | .71 | <.01 |  |  | .05 | .83 |
| TEQ05 |  | .88 | <.01 |  |  |  | .95 | <.01 |  | 1.03 | <.01 |  |  | .69 | .02 |
| TEQ06 |  | .94 | <.01 |  | 1.02 | <.01 |  |  |  | .90 | <.01 | .40 | .08 |  |  |
| TEQ07 |  | 1.26 | <.01 |  |  |  | 1.41 | <.01 |  | 1.39 | <.01 |  |  | .17 | .46 |
| TEQ08 |  | 1.24 | <.01 |  | 1.40 | <.01 |  |  |  | 1.18 | <.01 | .74 | <.01 |  |  |
| TEQ09 |  | 1.23 | <.01 |  | 1.36 | <.01 |  |  |  | 1.61 | .01 | 2.25 | .03 |  |  |
| TEQ10 |  | 1.51 | <.01 |  |  |  | 1.73 | <.01 |  | 1.85 | <.01 |  |  | .75 | .11 |
| TEQ11 |  | 1.01 | <.01 |  | 1.10 | <.01 |  |  |  | .89 | <.01 | .90 | <.01 |  |  |
| TEQ12 |  | .91 | <.01 |  |  |  | .98 | <.01 |  | 1.03 | <.01 |  |  | .66 | .03 |
| TEQ13 |  | 1.47 | <.01 |  | 1.75 | <.01 |  |  |  | 1.50 | <.01 | .38 | .09 |  |  |
| TEQ14 |  | 1.09 | <.01 |  |  |  | 1.20 | <.01 |  | 1.19 | <.01 |  |  | .13 | .58 |
| TEQ15 |  | 1.18 | .01 |  |  |  | 1.31 | .03 |  | 1.56 | .08 |  |  | -.46 | .34 |
| TEQ16 |  | .77 | <.01 |  | .83 | <.01 |  |  |  | .88 | <.01 | -.10 | .69 |  |  |
| TEQ17 |  | 1.05 | <.01 |  | 1.16 | <.01 |  |  |  | 1.01 | <.01 | .42 | .03 |  |  |
| TEQ18 |  | .85 | <.01 |  |  |  | .91 | <.01 |  | 1.10 | <.01 |  |  | -.46 | .18 |
| TEQ19 |  | .81 | <.01 |  |  |  | .87 | <.01 |  | .85 | <.01 |  |  | .28 | .23 |

**Figure S4.** Item characteristic and item information curves for TEQ items (n=532)


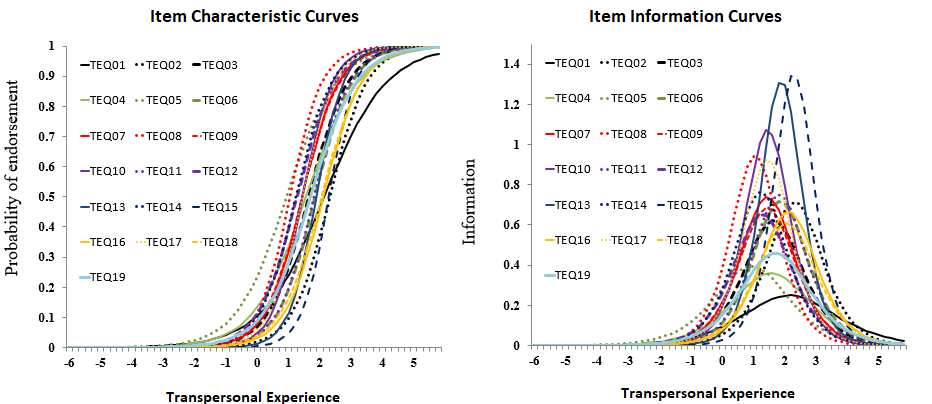


## **References**

Bentler, P. M. (1990). Comparative fit indexes in structural models. *Psychol Bull, 107*(2), 238-246. doi:10.1037/0033-2909.107.2.238

Bentler, P. M., & Bonett, D. G. (1980). Significance Tests and Goodness of Fit in the Analysis of Covariance-Structures. *Psychol Bull, 88*(3), 588-606. doi:Doi 10.1037/0033-2909.88.3.588

Browne, M. W., & Cudeck, R. (1993). Alternative ways of assessing model fit. In J. S. L. K.A. Bollen (Ed.), *Testing Structural Equation Models*. Newbury Park: Sage.

Cronbach, L. J. (1951). Coefficient alpha and the internal structure of tests. *Psychometrika, 16*(3), 297-334.

Hoelter, J. W. (1983). The Analysis of Covariance-Structures - Goodness-of-Fit Indexes. *Sociological Methods & Research, 11*(3), 325-344. doi:Doi 10.1177/0049124183011003003

Landis, J. R., & Koch, G. G. (1977). The measurement of observer agreement for categorical data. *Biometrics, 33*(1), 159-174. Retrieved from <https://www.ncbi.nlm.nih.gov/pubmed/843571>

Lord, F. M. (1980). *Applications of item response theory to practical testing problems*. Mahwah, NJ: Lawrence Erlbaum Associates, Inc.

Mason, O., Claridge, G., & Jackson, M. (1995). New Scales for the Assessment of Schizotypy. *Personality and Individual Differences, 18*(1), 7-13. doi:Doi 10.1016/0191-8869(94)00132-C

Muthén, B. O. (1984). A general structural equation model with dichotomous, ordered categorical, and continuous latent variable indicators. *Psychometrika, 49*(1), 115-132.

Viera, A. J., & Garrett, J. M. (2005). Understanding interobserver agreement: the kappa statistic. *Fam Med, 37*(5), 360-363. Retrieved from <https://www.ncbi.nlm.nih.gov/pubmed/15883903>

Wirth, R. J., & Edwards, M. C. (2007). Item factor analysis: current approaches and future directions. *Psychol Methods, 12*(1), 58-79. doi:10.1037/1082-989X.12.1.58
